# Editorial: Frontiers of women in brain imaging and brain stimulation

**DOI:** 10.3389/fnhum.2023.1208253

**Published:** 2023-05-17

**Authors:** Ruogu Fang, Lijun Bai, Wen Li

**Affiliations:** ^1^J. Crayton Pruitt Family Department of Biomedical Engineering, University of Florida, Gainesville, FL, United States; ^2^Department of Biomedical Engineering, School of Life Science and Technology, Xi'an Jiaotong University, Xi'an, Shaanxi, China; ^3^Department of Psychology, Florida State University, Tallahassee, FL, United States

**Keywords:** brain stimulation, women researchers, diversity and inclusion, neuroscience, neuroimaging

Advancements in brain imaging and stimulation have revolutionized our understanding of the human brain, its functioning, and the associated disorders. However, the historical male-dominated culture and practice in this field and the underrepresentation of women researchers and leaders could curb its development and limit its full potential. As such, this Research Topic, “*Women in brain imaging and stimulation*,” aims to showcase the work led by female researchers in the field and highlight their scholarship and scientific achievements at the frontier of interdisciplinary research of brain imaging and stimulation.

Rahman et al. combined depth-electrode stimulation and recordings in the mid-cingulate cortex (MCC) to achieve the precise localization of the grasping action. They found that a localized region in the mid-cingulate cortex elicited grasping action, which suggests that this region could be a target for motor neuromodulation treatment in specific movement disorders or neurorehabilitation. The study found that grasping action was elicited from a localized region in the MCC, providing evidence that the cingulate cortex's crucial involvement in the grasping network. This finding opens opportunities to explore the use of deep brain stimulation in this specific region as a motor neuromodulation target for specific movement disorders or neurorehabilitation.

Sun et al. combined electroencephalogram (EEG) and electroconvulsive therapy (ECT) to examine functional brain network changes associated with ECT remission in patients with major depressive disorder (MDD). The study found that ECT treatment significantly increased the global efficiency, edge betweenness centrality, local efficiency, and mean degree of the alpha band, which were associated with decreased depressive symptoms. This finding provides evidence to understand how ECT regulates the topology organization in functional brain networks of clinically remitted depressive patients.

Nissim et al. integrated magnetic resonance imaging (MRI) and high-definition transcranial direct current stimulation (HD-tDCS) to assess the relationship between baseline cortical volume and thickness and efficacy of HD-tDCS in patients with primary progressive aphasia (PPA). The study found that greater baseline thickness of the pars opercularis significantly predicted naming gains immediately following the intervention, while greater baseline volume of the left inferior frontal gyrus was associated with sustained gains for 6 weeks post-intervention. These findings provide insights into neural variations associated with different responses to neuromodulation and identify a useful biomarker for the potential use of cortical measures as predictors of tDCS-induced improvements in language abilities in PPA patients.

Herfurth et al. examined the use of magnetoencephalography (MEG) in pre-surgical language mapping for patients with pharmaco-resistant epilepsy. The study compared MEG with functional magnetic resonance imaging (fMRI) and validated the results using the Wada test. The researchers found that localizing the desynchronization of MEG beta power using a verb generation task is a promising tool for identifying language dominance in epilepsy patients. They also observed high agreement between MEG and Wada tests for language lateralization in inferior frontal, temporal, and parietal areas. They also suggested further research be performed due to the lower-than-expected overall agreement between MEG and fMRI.

Olsen et al. focused on the mechanisms and factors involved in memory enhancement through vagus nerve stimulation (VNS). The authors discussed the clinical and preclinical studies indicating that VNS can improve memory and highlighted the involvement of brain regions such as the prefrontal cortex and processes such as alertness and arousal. They also emphasized the importance of targeting VNS to specific brain areas, timing in relation to training, and considering sex-specific factors to optimize its application for enhancing memory. Overall, the study provides insights into the potential benefits of VNS for improving memory and the need for a better understanding of its mechanisms and optimal administration.

These contributions exhibit the cutting-edge, sophisticated scientific investigations led by women scientists at the interface of neuroimaging and neuromodulation. These studies also demonstrate how promoting representation across sex, gender, and race can advance brain imaging and stimulation research to ensure that findings are applicable across diverse populations. In conclusion, women scientists are a vital force in advancing the field of brain imaging and stimulation. It is crucial that we continue to support and promote diversity and particularly the female gender in this field and neuroscience in general. By creating a leveled platform across genders, we can not only foster a more inclusive and diverse research community but also actualize its full talents and prowess, which will ultimately lead to better outcomes for patients.

## Author contributions

All authors listed have made a substantial, direct, and intellectual contribution to the work and approved it for publication.

